# Genetic, Epigenetic and Environmental Factors Influence the Phenotype of Tooth Number, Size and Shape: Anterior Maxillary Supernumeraries and the Morphology of Mandibular Incisors

**DOI:** 10.3390/genes13122232

**Published:** 2022-11-28

**Authors:** Khaled Khalaf, Alan Henry Brook, Richard Nigel Smith

**Affiliations:** 1 Institute of Dentistry, University of Aberdeen, Aberdeen AB25 2ZR, UK; 2 School of Dentistry, The University of Adelaide, Adelaide, SA 5000, Australia; 3 School of Dentistry, University of Liverpool, Liverpool L3 5PS, UK

**Keywords:** mandibular incisor morphology, supernumerary teeth, principal components, many models, genetic interactions

## Abstract

The aim of this study is to investigate whether the genetic, epigenetic and environmental factors that give rise to supernumeraries in the maxillary incisor region and larger dimensions of the adjacent maxillary incisors are also associated with variations in the morphology of the mandibular incisors. If so, this would contribute to understanding the distribution and interactions of factors during dental development and how these can be modelled. The sample consisted of 34 patients with supernumerary teeth in the maxillary anterior region, matched for gender, age and White Caucasian ethnicity with 34 control subjects. The average ages of the supernumerary and control groups were 12.8 and 12.2 years, respectively. Study models of all subjects were constructed and imaged using a previously validated system. Using custom software, each of the mandibular incisor teeth were measured to obtain 17 parameters from the labial view and 17 from the occlusal view. Principal component analysis (PCA) was used to summarize the measurements into a smaller set representing distinct features of the clinical crowns, followed by a comparison between the supernumerary and control groups using 2-way ANOVA. Seven factors of tooth size of the mandibular central incisors and six factors of the mandibular lateral incisors were identified as major features of the clinical crowns. All parameters of both mandibular incisors were greater in the supernumerary group than in the control, with three of these, located in the incisal and cervical regions of the mandibular lateral incisors, being statistically significantly larger. The findings of this study indicate that the aetiological factors associated with supernumerary teeth in the maxillary anterior region also affect tooth crown dimensions of mandibular incisors. This new evidence enhances several models of the interactions of genetic, epigenetic and environmental components of dental development and supports a multi-model approach to increase understanding of this process and its variations.

## 1. Introduction

Dental development is multifactorial with interactions between genetic, epigenetic and environmental factors at multiple stages during the process. The phenotypic outcome includes variation in tooth number, size and shape. These variations often involve only the dentition, but are sometimes part of a syndrome. Supernumerary teeth are common dental variations in humans with a prevalence ranging from 0.1% to 3.8% in different populations and are more frequent in the permanent than the primary dentition [1,2,3,4]. They are more common in males than females, which is in contrast to the gender difference of the prevalence of hypodontia [1,5,6].

The aetiology of supernumerary teeth is multifactorial, involving complex interactions as the dentition develops within the craniofacial complex [5,6,7,8,9,10,11,12,13,14,15,16]. Genetic factors associated with supernumerary teeth identified in the mouse dentition include mutations of Fgf, Eda, Osr2, Runx2, Apc, Shh and Wnt/b-catenin [17,18,19,20,21]. Human patients with supernumerary teeth show enhanced expression of WNT and SHH proteins as well as reduced expression of APC protein, indicating derangement of molecular pathways [22]. In an assessment of risk factors and molecular biomarkers in children with supernumerary teeth Talaat et al. [22], found that patients with supernumerary teeth not only had increased expression of WNT and SHH proteins and reduced expression of APC protein but also had epigenetic and environmental factors involved. A history of severe oral infection, a medical history of chemotherapy, a maternal history of medication or illness during pregnancy, a family history of neoplastic disorders, the use of electronic devices, and living beside agricultural fields or industrial areas were statistically significantly associated with an increased risk of supernumerary tooth development [22].

Supernumerary teeth occur in different areas of the dentition with the most common location being the maxillary anterior region [1,23,24,25,26,27,28]. Khalaf et al., [29] showed that the presence of supernumerary teeth in any location of the maxillary or mandibular dental arches was associated with a tendency to have permanent teeth of larger sizes than controls. A further study by Khalaf et al. [30] has investigated the impact of supernumerary teeth in only the maxillary anterior region on crown size measurements of the adjacent permanent incisors. This study found that the adjacent incisors had larger tooth sizes than controls; the central incisors were more affected than the lateral incisors, suggesting a local Morphological Field effect [31]. However, there is no published investigation on whether the presence of supernumerary teeth in the maxillary anterior region is also associated with larger crown dimensions of the mandibular incisors.

Brook and Brook O’Donnell [32] have applied recent advances in Complexity Science [33], Network Science [34] and Many-Model Thinking [35] to studying the development and emergent phenotypic outcome of the dentition and the dental arches. In this paper, this approach is applied to interpreting the findings of studies of supernumerary teeth in the maxillary anterior region and the related incisor teeth. This will enhance understanding of the process of dental development and its phenotypic outcomes. In addition, since the premaxilla is the most frequent site for supernumerary teeth in humans and these often disturb the eruption and alignment of other teeth, basic science investigations about the possible morphological differences in the incisors will have impacts for clinical treatment planning.

Therefore, the aim of this study is to investigate whether the aetiological factors that give rise to supernumeraries in the maxillary incisor region and larger dimensions of the adjacent maxillary incisors also associated with variations in the morphology of the mandibular incisors. Interpreting these results will contribute to increasing understanding of the interactions of factors during development and how they can be modelled.

## 2. Materials and Methods

This is an analytical cross sectional study comparing tooth size measurements in subjects with supernumerary teeth in the maxillary anterior region and a control group. Ethical approval was granted by the South Yorkshire Ethics Committee, UK (South Sheffield Research Ethics Committee Number 98/354) prior to conducting this study. A sample size power calculation was carried out under the guidance of the Statistical Department, University of Sheffield, to identify the required number of cases and controls based on 0.05 α, 0.80 β and 1.5 mm clinically significant difference between the groups’ means of the mesio-distal or bucco-lingual dimensions. This power calculation found that 15 individuals would be required in each subgroup. Additional supernumerary patients and controls were included in case it was not possible to take all of the measurements for each individual.

The dental register of patients attending the orthodontic and pediatric dentistry clinics at Charles Clifford Dental Hospital, Sheffield, UK was searched to identify subjects with supernumerary teeth in the maxillary anterior region. The inclusion criteria for patients were: white Caucasians, with no general medical conditions, syndromes or significant skeletal discrepancies. The patients were age- and gender- matched with controls from the same dental register. The sample consisted of 34 subjects with supernumerary teeth (17 males and 17 females), age- and gender-matched with 34 control subjects (17 males and 17 females). All supernumerary teeth were located in the maxillary incisor region and the majority of patients had one or two supernumeraries. The average age of the supernumerary and control groups was 12.8 and 12.2 years, respectively.

**Figure 1:**
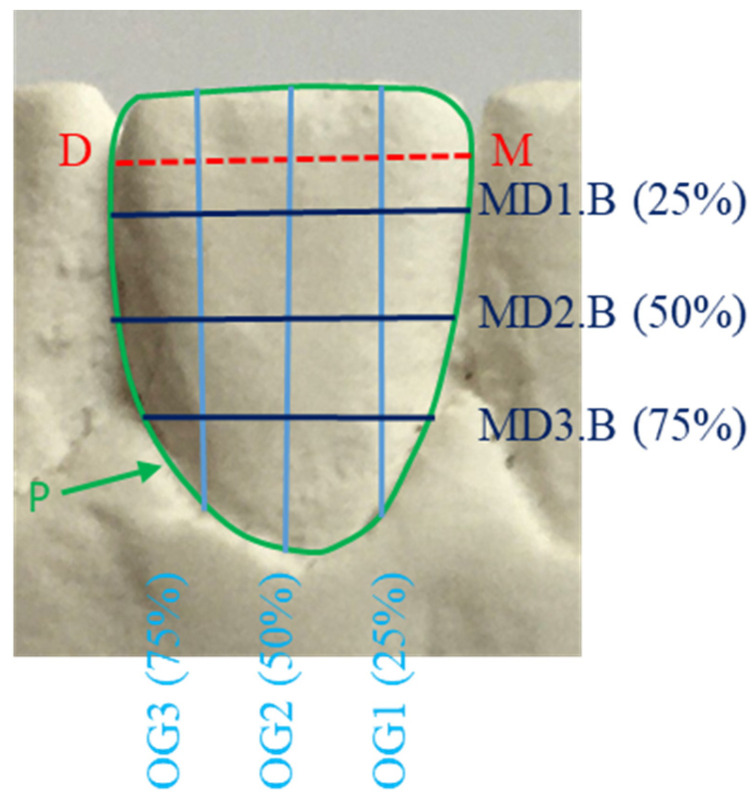
Buccal view of a lower right central incisor measurements: MD: maximal mesio-distal dimension at the contact points; MD1.B, MD2.B, MD3.B are mesio-distal dimensions at 25, 50 and 75% along a line bisecting the MD, respectively; OG1, OG2, OG3 occluso-gingival dimensions, at 25, 50 and 75% along the MD, respectively; P is tooth surface perimeter.

**Figure 2:**
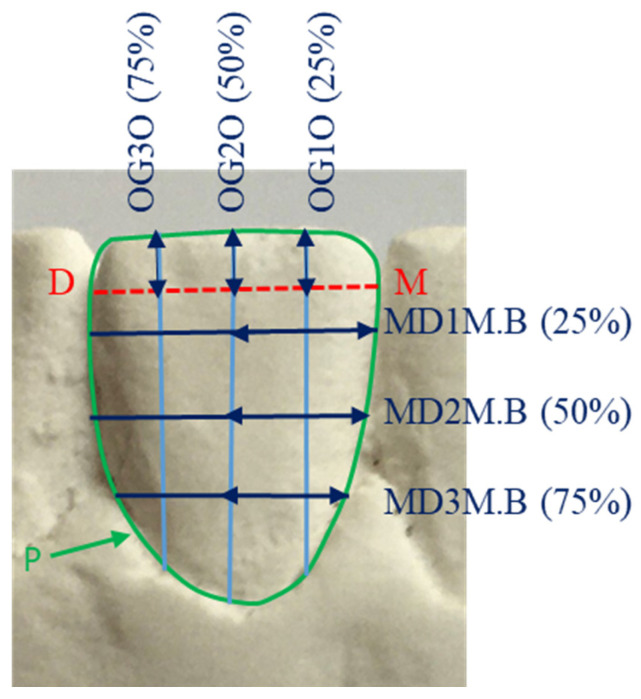
Buccal view of a lower right central incisor partial measurements: MD1M.B, MD2M.B and MD3M.B are the mesial parts of the mesio-distal measurements at 25, 50 and 75% along the OG2, respectively and determined by OG2; OG1O, OG2O and OG3O are the occlusal parts of the occluso-gingival measurements at 25, 50 and 75% along the MD, respectively and determined by the MD.

**Figure 3:**
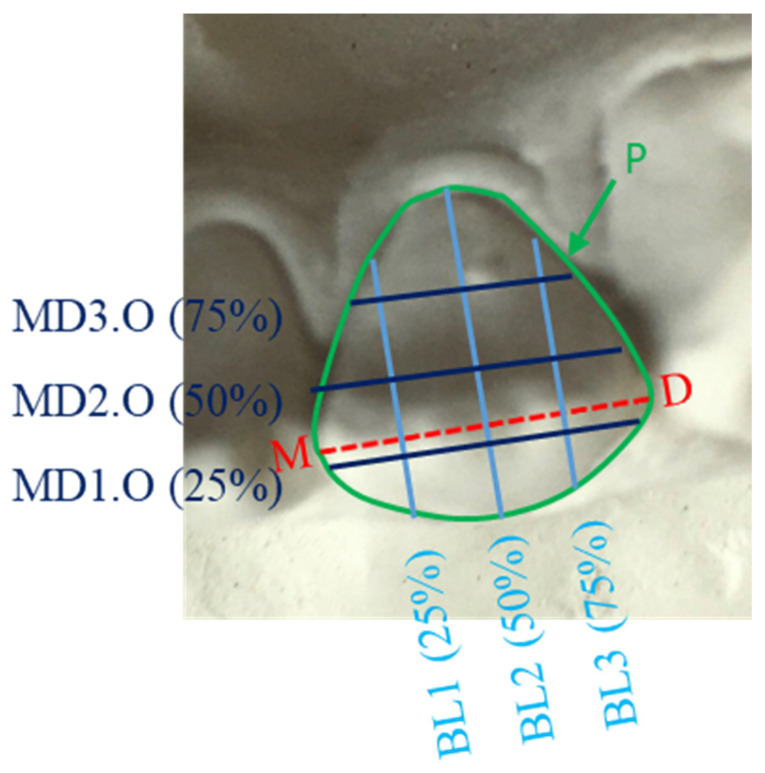
Occlusal view of a lower left lateral incisor measurements: BL1, BL2, BL3 are bucco-lingual dimensions at 25, 50 and 75% along the MD, respectively. MD1.O, MD2.O and MD3.O are mesio-distal dimensions at 25, 50 and 75% along a line bisecting the MD.

**Figure 4:**
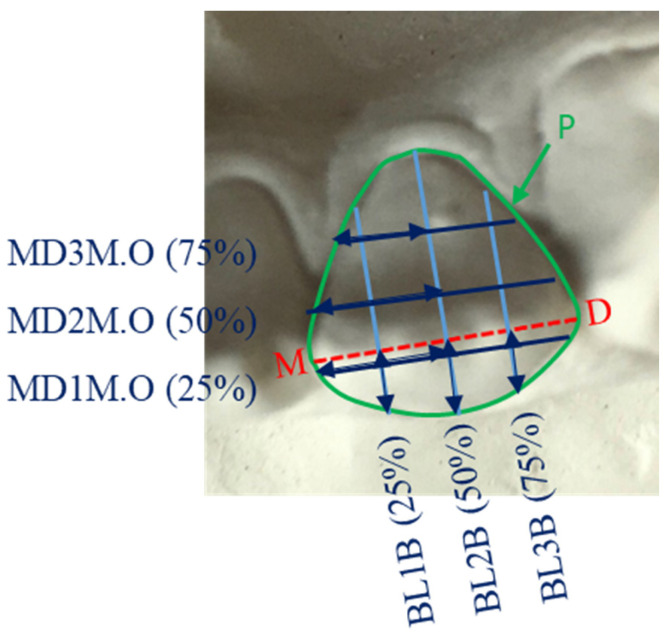
Occlusal view of a lower left lateral incisor partial measurements: MD1M.O, MD2M.O and MD3M.O are the mesial parts of the mesio-distal measurements at 25, 50 and 75% along BL2, respectively and determined by BL2; BL1B, BL2B and BL3B are the buccal parts of the bucco-lingual measurements at 25, 50 and 75% along the MD, respectively and determined by the MD.

Informed consent was obtained from all subjects. Study models of all subjects were constructed using standardized methods and the mandibular incisor teeth were imaged and measured using 2D image analysis techniques described in the previous studies [29,30]. Teeth that were carious, severely crowded, with large restorations, excessive tooth wear, overgrowth or severe recession were excluded. Seventeen variables from the labial view (Table 1 and Figure 1 and Figure 2) and 17 variables from the occlusal view (Table 2 and Figure 3 and Figure 4) of each mandibular incisor were measured using custom-made software.

Measurements taken from the labial view and bounded by the periphery of the tooth were: the standard mesio-distal and occluso-gingival dimensions, as well as additional occluso-gingival dimensions at 25 and 75% of the mesio-distal line; and additional mesio-distal dimensions at 25, 50 and 75% of the occluso-gingival diameter (Figure 1 and Figure 2). Similarly, from the occlusal view the measurements were: the standard mesio-distal and labio-lingual dimensions, as well as additional labio-lingual dimensions at 25 and 75% of the mesio-distal line; and additional mesio-distal dimensions at 25, 50 and 75% of the labio-lingual diameter (Figure 2 and Figure 4).

Measurements from the right and left mandibular incisors were averaged as there were no significant differences in the variance between right and left sides within patients as compared to the variance between patients using intra-class correlation coefficients. Principal component analysis (PCA) was used to summarize the measurements and reduce the number of variables to a meaningful number of key factor variables representing the measured dimensions likely to show tooth size variance relationships and differences. The resultant final factor variables were subsequently compared between groups and genders using 2-way ANOVA.

## 3. Results

### 3.1. Results of Principal Component Analysis

#### 3.1.1. Mandibular Central Incisors

**Figure 5:**
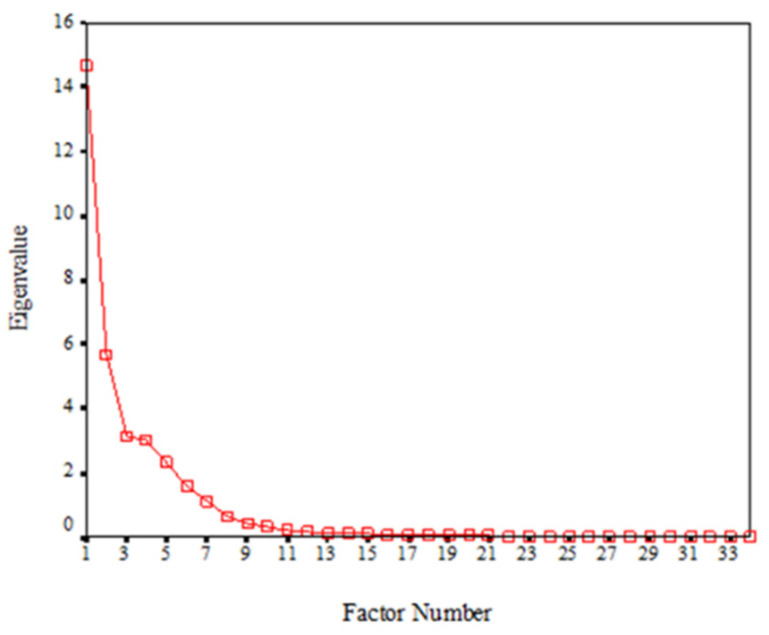
Scree plot, before rotation, for factor extraction of mandibular central incisor size variables in the supernumerary group.

As shown in Figure 5 and Table 3, the first seven factors together accounted for most of the variance in the dataset (92.7% of the total variance of the mandibular central incisor measurements) and these were extracted as representative variables of tooth size. Each factor has multiple variables with high variance loadings on it and all share a common feature. A factor score was calculated based on the various components making up the factor. The identified seven factors were as follows:

- Factor 1: The large component of tooth length (LCOTL). Factor score = average (MD.B, MD.O).
- Factor 2: Tooth size from the occlusal view (TSFOV). Factor score = A.O.
- Factor 3: Tooth size from the buccal view (TSFBV). Factor score = A.B.
- Factor 4: The buccal component of tooth size from the occlusal view (BCOTSFOV). Factor score = AB.O.
- Factor 5: The occlusal component of tooth size from the buccal view (OCOTSFBV). Factor score = AO.B.
- Factor 6: The small component of tooth length from the occlusal view (SCOTLFOV). Factor score = MD3.O.
- Factor 7: The small component of tooth length from the buccal view (SCOTLFBV). Factor score = MD3.B.

Factor 1 above has the greatest contribution to tooth size (accounted for 27.3% of the variability in tooth size), whereas factor 7 has the least contribution (6.6 % of the variability in tooth size) (Table 3).

**Table 3:** Total and percent variance of the first seven factors for mandibular central incisor size variables in the supernumerary group. Note: The ‘‘Rotation Sums of Squared Loadings’’ shows factor variance after rotation (Varimax) which was used to transform the initial matrix into one that is easier to interpret.

Factor	Rotation Sums of Squared Loadings		
	Total	% of Variance	Cumulative %
**1**	9.29	27.34	27.34
**2**	5.18	15.24	42.58
**3**	4.31	12.68	55.26
**4**	4.03	11.85	67.11
**5**	3.91	11.51	78.62
**6**	2.56	7.53	86.15
**7**	2.24	6.58	92.74

#### 3.1.2. Mandibular Lateral Incisors

**Figure 6:**
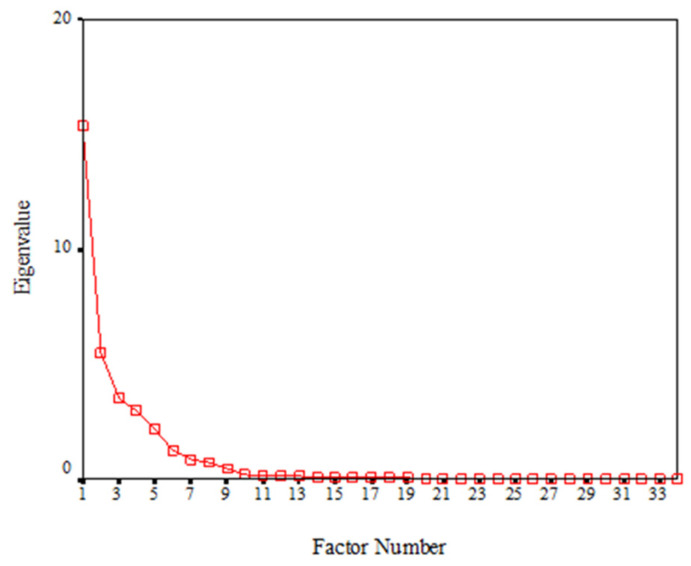
Scree plot, before rotation, for factor extraction of mandibular lateral incisor size variables in the supernumerary group.

As shown in Figure 6 and Table 4, the first six factors together accounted for most of the variance in the dataset (90.4% of the total variance of the mandibular lateral incisor measurements) and thus were extracted as representative variables of tooth size. The identified six factors were as follows:

- Factor 1: The large component of tooth length (LCOTL). Factor score = average (MD.B, MD.O).
- Factor 2: Tooth size from the occlusal view (TSFOV). Factor score = A.O.
- Factor 3: Tooth size from the buccal view (TSFBV). Factor score = A.B.
- Factor 4: The occlusal component of tooth size from the buccal view (OCOTSFBV). Factor score = AO.B.
- Factor 5: The buccal component of tooth size from the occlusal view (BCOTSFOV). Factor score = AB.O.
- Factor 6: the small component of tooth length (SCOTL). Factor score = MD3.O.

Factor 1 above has the greatest contribution to tooth size (accounted for 27.0% of the variability in tooth size), whereas factor 6 has the least contribution (10.5% of the variability in tooth size) (Table 4).

**Table 4:** Total and percent variance of the first six factors for mandibular lateral incisor size variables in the supernumerary group. Note: The ‘‘Rotation Sums of Squared Loadings’’ shows factor variance after rotation (Varimax) which was used to transform the initial matrix into one that is easier to interpret.

Factor	Rotation Sums of Squared Loadings		
	Total	% of Variance	Cumulative %
**1**	9.19	27.02	27.02
**2**	5.47	16.07	43.09
**3**	4.95	14.55	57.64
**4**	3.81	11.21	68.85
**5**	3.76	11.05	79.90
**6**	3.57	10.51	90.41

### 3.2. Results of Comparisons between the Supernumerary and Control Groups

Table 5 and Table 6 show results of the comparison study of size variables mentioned above between the supernumerary and control groups for the mandibular central and lateral incisors, respectively.

The range of the mean difference between the supernumerary group and control for the central incisors was 0.06–0.32 mm and 0.04–1.58 mm^2^ for lineal and tooth surface area measurements, respectively; and for the lateral incisors 0.03–0.35 mm and 0.08–1.93 mm^2^, respectively. Whilst none of the differences were significant (*p* < 0.05) for the mandibular central incisors, three variables had significant differences for the mandibular lateral incisors. These were: LCOTL in males only, OCOTSFBV and SCOTL.

## 4. Discussion

The present results extend the findings of Khalaf et al. [30] who investigated the association between the presence of supernumerary teeth in the maxillary anterior region and tooth size variation of the maxillary incisors. Here, we investigate whether supernumerary teeth in the maxillary anterior region are also associated with crown size variation of teeth of the same morphological class of the adjacent incisors, but in the opposing dental arch. These new findings increase understanding of the distribution of the multiple factors interacting at different sites during dental development and provide further knowledge to apply to the different models of dental development.

The finding that all variables of the mandibular incisor teeth were larger in the supernumerary group than in the control, although only a few reached the 0.05 significance level (LCOTL, OCOTSFBV and SCOTL), is similar to a previous study that investigated multiple image analysis measurements of tooth size (linear, perimeter and tooth surface areas) in patients with supernumerary teeth in all locations around the dental arches compared with a control group [29]. Furthermore, similar results were reported with regard to crown size variation of the maxillary incisors associated with the presence of supernumerary teeth in the maxillary anterior region using the same methods and measurements of the current study [30]. These findings from the three studies by Khalaf et al. [29,30], the present study) indicate that the aetiological factors that contributed to the occurrence of supernumerary teeth in the maxillary anterior region had a greater impact on development of the adjacent teeth and also on mandibular incisor teeth than on the posterior teeth suggesting a regional effect.

Since all permanent incisors in both dental arches develop within a limited time interval [36], genetic, epigenetic and environmental factors will be involved in the complex interactions leading to these variations in tooth number, size and shape. In this regard, Dempsey and Townsend [37], showed that different teeth within each morphological class of the human dentition share similar heritabilities with regard to variation in the mesiodistal (MD) and buccolingual (BL) crown diameters. Moreover, the detailed measurements of the mandibular central and lateral incisors and the outcome of the principal component analysis of summarizing these measurements into a smaller set of characteristic features of tooth morphology shows that both mandibular incisors share a similar morphology in contrast to the maxillary incisors. In addition, it was found that the mandibular lateral incisors were more variable anatomically than the mandibular central incisors in the supernumerary group patients when compared with controls. Three factor variables of the mandibular lateral incisors were found to be significantly greater in the supernumerary group (LCOTL, OCOTSFBV and SCOTL) compared with none of the mandibular central incisors. This may be due to the smaller size of the mandibular central incisor together with it being more symmetrical than the mandibular lateral incisor and less variable morphologically than the mandibular lateral incisor [38].

Although, all linear measurements were greater in males than females across both the supernumerary and control groups, none of the differences were statistically significant (*p* < 0.05). This agrees with previous studies on differences between sexes with regard to the mesio-distal and labiolingual measurements in the normal human dentition [30,39,40,41,42]. This may mainly be due to the relatively longer period of dental development in males when compared with females [43].

The new findings from this study together with those on the same sample by Khalaf et al. [29,30], demonstrate a pattern of variation across the dentition involving tooth number, size and shape, which can be explored to increase understanding of the complex interacting networks containing multiple factors during dental development. How these new findings relate to different models proposed for dental development will now be considered. These models can be grouped under those emphasizing phenotypic presentations, patterning within the dentition, and molecular genetics.

**Figure 7:**
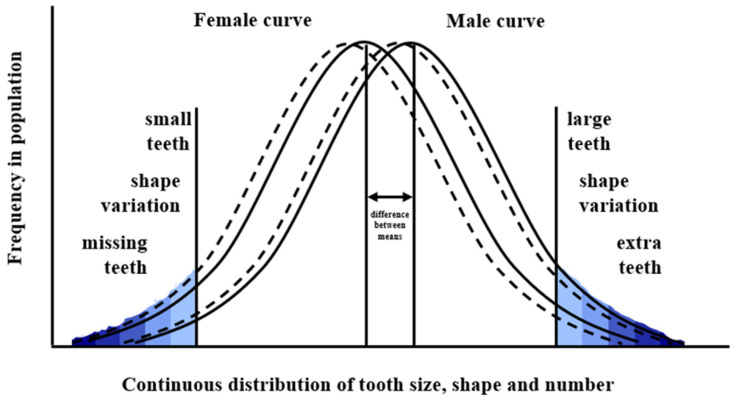
Model of the normal distribution of tooth number, size and shape with thresholds beyond which clinically relevant variations occur. The variation in the shading reflects the increasing severity as the tails of the distribution are approached. The dotted curves are for female and male Romano-Britons., showing smaller teeth probably related to severe environmental insults [11].

In models based on the phenotype the findings here are compatible with the quasi-continuous model based on the normal distribution of tooth number and size developed by Brook [5], and further developed to include tooth shape [11,32]. This is a random network model (Figure 7). In addition, the distributions shown in Figure 5 and Figure 6 for the lower incisors and in the Khalaf’s [30] study, for the upper incisors are compatible with power law distributions, which are found in complex systems [33]. 

Patterning models concerning the dentition are also relevant to these results. Morphogenetic field theory applied to the mammalian dentition by Butler, [31], postulates that teeth develop in a specific manner in their morphological fields, such that the teeth further away from the center of each field are more variable than the key tooth. Applying Butler’s concept to the human dentition, Dahlberg [44] considered the maxillary central incisor, mandibular lateral incisor, canine, first premolar and first molar to be the most stable and key teeth in their respective morphological classes. The findings in this study that the significant differences in the mandibular incisor measurements of the supernumerary patients were towards the incisal edge and cervical region, suggest that, while the etiological factors of the supernumerary teeth affect the crown morphology of the mandibular incisors throughout their development, there is a greater impact at the early and late stages of crown formation. This is supported by the results of the previous investigation of crown size variation of the maxillary permanent incisors in patients with supernumerary teeth in the maxillary anterior region [30].

**Figure 8:**
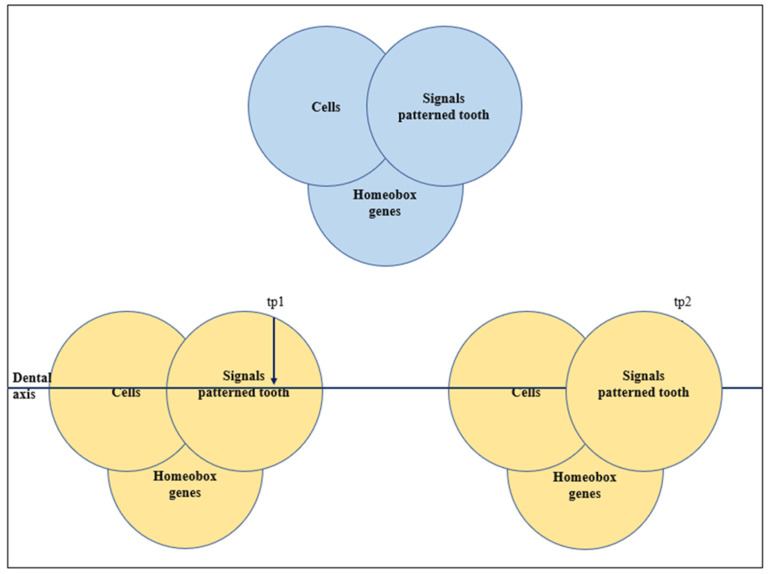
Co-operative genetic interaction (CGI) model (derived from Mitsiadis & Smith (2006) [46]) tp1 refers to tooth position 1, tp2 refers to tooth position 2.

A model based on the molecular genetics of tooth development in mice is the homeobox model proposed by Sharpe [45], which suggests that tooth morphology is the result of varying expression on several homeobox genes in ectomesenchymal cells. Mitsiadis and Smith [46] suggested a co-operative genetic interaction model that incorporated the homeobox gene model, morphogenetic fields and the Osborne’s [47] clone theory (Figure 8). According to this concept every component, including cells and homeobox genes is acting to pattern teeth. This combination of models was further explored by Townsend et al. [48].

No one model can fully encompass the multiple dimensions and components of dental development. Rather each model contributes to understanding of the phenotypic variations of the patterning and the interactions that occur during development of the human dentition.

It is important to interpret the above findings within the limitations of the current study. Due to the rarity of the prevalence of more than two supernumerary teeth in the maxillary anterior region, it was not possible to investigate the severity of the condition and the type of supernumerary tooth on crown size variation of the lower incisors. Additionally, it may be argued that it would have been better to account for dental arch dimensions to eliminate the possibility of introducing a confounding variable.

## 5. Conclusions

Seven factors were identified as distinctive features of the clinical crowns of the mandibular central and six of the lateral incisors. Although all variables of both incisors were greater in the supernumerary group when compared with controls, only three of these, located in the incisal and cervical regions, and only for the mandibular lateral incisors, reached the 0.05 significance level. The new findings contributed by this study suggest that the aetiological factors associated with supernumerary teeth in the maxillary anterior region also affect tooth crown dimensions of mandibular incisors. This new evidence supports a multi-model approach to increase understanding of the process and the variation of dental development.

## Figures and Tables

**Table 1:** Variables measured from the buccal view of each tooth.

Measurement Code	Measurement Description
1. P.B	The perimeter of the tooth surface
2. A.B	The tooth surface area bounded by the perimeter trace
3. MD.B	The maximum distance between the contact areas of the proximal surfaces
4. OG1.B	The length perpendicular to and at 25% along MD.B, from the mesial aspect
5. OG1O.B	The portion of OG1.B, limited by the occlusal aspect and MD.B
6. OG2.B	The length perpendicular to and at 50% along MD.B
7. OG2O.B	The portion of OG2.B, limited by the occlusal aspect and MD.B
8. OG3.B	The length perpendicular to and at 75% along MD.B, from the mesial aspect
9. OG3O.B	The portion of OG3.B, limited by the occlusal aspect and MD.B
10. MD1.B	The length perpendicular to and at 25% along OG2.B, from the occlusal aspect
11. MD1M.B	The length of MD1.B, bounded by the mesial aspect and OG2.B
12. MD2.B	The length perpendicular to and at 50% along OG2.B
13. MD2M.B	The length of MD2.B, bounded by the mesial aspect and OG2.B
14. MD3.B	The length perpendicular to and at 75% along OG2.B from the occlusal aspect
15. MD3M.B	The length of MD3.B, bounded by the mesial aspect and OG2.B
16. AO.B	Part of the total area (A.B), bounded by MD.B and positioned occlusally
17. AM.B	Part of the total area (A.B), bounded by OG2.B and positioned mesially

**Table 2:** Variables measured from the occlusal view of each tooth.

Measurement Code	Measurement Description
1. P.O	The perimeter of the tooth surface
2. A.O	The tooth surface area bounded by the perimeter trace
3. MD.O	The maximum distance between the contact areas of the proximal surfaces
4. BL1.O	The length perpendicular to and at 25% along MD.O, from the mesial aspect
5. BL1B.O	The portion of BL1.O, limited by the buccal aspect and MD.O
6. BL2.O	The length perpendicular to and at 50% along MD.O
7. BL2B.O	The portion of BL2.O, limited by the buccal aspect and MD.O
8. BL3.O	The length perpendicular to and at 75% along MD.O, from the mesial aspect
9. BL3B.O	The portion of BL3.O, limited by the buccal aspect and MD.O
10. MD1.O	The length perpendicular to and at 25% along BL2.O, from the buccal aspect
11. MD1M.O	The length of MD1.O, bounded by the mesial aspect and BL2.O
12. MD2.O	The length perpendicular to and at 50% along BL2.O
13. MD2M.O	The length of MD2.O, bounded by the mesial aspect and BL2.O
14. MD3.O	The length perpendicular to and at 75% along BL2.O from the buccal aspect
15. MD3M.O	The length of MD3.O, bounded by the mesial aspect and BL2.O
16. AB.O	Part of the total area (A.O), bounded by MD.O and positioned buccally
17. AM.O	Part of the total area (A.O), bounded by BL2.O and positioned mesially

**Table 5:** Variables of mandibular central incisors of supernumerary and control groups.

Variable		Supernumerary			Control		
		N1	Mean (mm)/(mm)^2^	S.D. (mm)/(mm)^2^	N2	Mean (mm)/(mm)^2^	S.D. (mm)/(mm)^2^
LCOTL	F	17	5.60	0.35	17	5.54	0.28
	M	17	5.79	0.36	17	5.47	0.31
	Total	34	5.70	0.36	34	5.51	0.29
TSFOV	F	17	24.76	4.31	17	23.50	2.12
	M	17	26.33	3.31	17	24.85	2.73
	Total	34	25.55	3.87	34	24.21	2.49
TSFBV	F	17	35.09	4.53	17	33.96	4.19
	M	17	37.46	4.45	17	35.88	4.23
	Total	34	36.28	4.59	34	35.00	4.27
BCOTSFOV	F	17	7.34	1.62	17	7.30	0.92
	M	17	7.68	1.70	17	7.64	1.50
	Total	34	7.51	1.65	34	7.48	1.22
OCOTSFBV	F	17	8.60	1.57	17	7.07	1.90
	M	17	8.63	2.37	17	7.33	1.75
	Total	34	8.62 *	1.99	34	7.19 *	1.81
SCOTLFOV	F	17	3.24	0.37	17	3.13	0.36
	M	17	3.31	0.35	17	3.16	0.42
	Total	34	3.28	0.35	34	3.14	0.38
SCOTLFBV	F	17	3.80	0.53	17	3.62	0.43
	M	17	4.02	0.63	17	3.82	0.39
	Total	34	3.91	0.59	34	3.72	0.42

Key: N1 = number of teeth measured from individuals with supernumeraries; N2 = number of teeth measured from control subjects; M = males; F = females. S.D. = standard deviation. * *p* < 0.05 (Bonferroni adjusted significance level accounting for multi-group comparisons and multiple variables testing).

**Table 6:** Variables of mandibular lateral incisors of supernumerary and control groups.

Variable		Supernumerary			Control		
		N1	Mean (mm)/(mm)^2^	S.D. (mm)/(mm)^2^	N2	Mean (mm)/(mm)^2^	S.D. (mm)/(mm)^2^
LCOTL	F	17	5.96	0.36	17	5.93	0.21
	M	17	6.35 *	0.44	17	6.00 *	0.39
	Total	34	6.16	0.44	34	5.97	0.31
TSFOV	F	17	28.71	3.86	17	26.93	2.23
	M	17	29.54	3.40	17	28.37	3.62
	Total	34	29.12	3.61	34	27.67	3.04
TSFBV	F	17	35.15	4.92	17	35.23	3.88
	M	17	37.04	5.03	17	35.68	3.96
	Total	34	36.12	5.00	34	35.46	3.87
OCOTSFBV	F	17	10.67 *	2.61	17	8.74 *	1.80
	M	17	12.11 *	2.52	17	9.61 *	2.07
	Total	34	11.41 *	2.63	34	9.19 *	1.97
BCOTSFOV	F	17	8.76	1.75	17	7.88	0.88
	M	17	8.64	1.69	17	8.47	1.39
	Total	34	8.70	1.70	34	8.18	1.18
SCOTL	F	17	3.62 *	0.32	17	3.40 *	0.35
	M	17	3.91 *	0.50	17	3.64 *	0.38
	Total	34	3.76 *	0.44	34	3.52 *	0.38

Key: N1 = number of teeth measured from individuals with supernumeraries; N2 = number of teeth measured from control subjects; M = males; F = females. S.D. = standard deviation. * *p* < 0.05 (Bonferroni adjusted significance level accounting for multi-group comparisons and multiple variables testing).

## Data Availability

Not applicable.
